# Impact of MRI resolution for Linac-based stereotactic radiosurgery

**DOI:** 10.3389/fonc.2023.1090582

**Published:** 2023-01-24

**Authors:** Yimei Huang, Evan Liang, Eric M. Schaff, Bo Zhao, Karen C. Snyder, Indrin J. Chetty, Mira M. Shah, Salim M. Siddiqui

**Affiliations:** Department of Radiation Oncology, Henry Ford Health, Detroit, MI, United States

**Keywords:** MRI resolution, partial volume, radiosurgery, target definition, plan quality

## Abstract

**Objective:**

Magnetic resonance imaging (MRI) is a standard imaging modality in intracranial stereotactic radiosurgery (SRS) for defining target volumes. However, wide disparities in MRI resolution exist, which could directly impact accuracy of target delineation. Here, sequences with various MRI resolution were acquired on phantoms to evaluate the effect on volume definition and dosimetric consequence for cranial SRS.

**Materials/Methods:**

Four T1-weighted MR sequences with increasing 3D resolution were compared, including two Spin Echo (SE) 2D acquisitions with 5mm and 3mm slice thickness (*SE5mm, SE3mm*) and two gradient echo 3D acquisitions (*TFE, BRAVO*). The voxel sizes were 0.4×0.4×5.0, 0.5×0.5×3.0, 0.9×0.9×1.25, and 0.4×0.4×0.5 mm^3^, respectively. Four phantoms with simulated lesions of different shape and volume (range, 0.53–25.0 cm^3^) were imaged, resulting in 16 total sets of MRIs. Four radiation oncologists provided contours on individual MR image set. All observer contours were compared with ground truth, defined on CT image according to the absolute dimensions of the target structure, using Dice similarity coefficient (*DSC*), Hausdorff distance (*HD*), mean distance-to-agreement (*MDA*), and the ratio between reconstructed and true volume (*Ratio_vol_*). For dosimetric consequence, SRS plans targeting observer volumes were created. The true Paddick conformity index ( $CI_{paddick}^{true}$ ), calculated with true target volume, was correlated with quality of observer volume.

**Results:**

All measures of observer contours improved as increasingly higher MRI resolution was provided from *SE5mm* to *BRAVO*. The improvement in *DSC*, *HD* and *MDA* was statistically significant (p<0.01). Dosimetrically, $CI_{paddick}^{true}\;$ strongly correlated with *DSC* of the planning observer volume (Pearson’s r=0.94, p<0.00001).

**Conclusions:**

Significant improvement in target definition and reduced inter-observer variation was observed as the MRI resolution improved, which also improved the quality of SRS plans. Results imply that high resolution 3D MR sequences should be used to minimize potential errors in target definition, and multi-slice 2D sequences should be avoided.

## 1 Introduction

Single- and multi-fraction stereotactic radiosurgery (SRS) has long been established as an effective treatment option for metastatic or primary brain tumors (1–8). It has also been applied successfully in treating benign conditions such as trigeminal neuralgia (9) and movement disorders (10). Due to the various indications for SRS, the size of the target volume can vary from millimeters to a few centimeters in diameter. Accurate delineation of the target volume for SRS treatments is crucial. Underrepresentation of the true target volume may lead to decreased tumor control probability. On the other hand, overrepresentation of target volume could unnecessarily increase the normal tissue complication probability due to surrounding organs-at-risk (11, 12).

Because of the superior soft tissue contrast afforded by MRI, T1-weighted contrast-enhanced MRI is the standard imaging modality for delineation of target volumes in SRS. High-resolution MRI scans are often ordered specifically for improving contouring accuracy (13, 14). A recent consensus recommendation for brain metastases imaging specifies post-contrast T1-weighted images using either a 3D pulse sequence with ≤ 1 mm isotropic resolution obtained in a 3T MR scanner, or ≤ 1.5 mm isotropic resolution obtained in a 1.5T scanner (15). Because of the higher spatial resolution, 3D pulse sequences are normally preferred over multi-slice 2D sequences. However, due to logistic or economic constraints, diagnostic MRIs for radiologic assessment are sometimes utilized for treatment planning. The diagnostic MRIs are often acquired using multi-slice 2D sequences and optimized for speed or other considerations, rather than the spatial resolution and geometric accuracy.

Existing literature on the variability of target volume measurement due to MR protocol variations is limited. Studies have shown that thin-slice MR imaging protocols can reveal an increased number of brain metastases (16, 17). Snell et al. simulated the effect with a synthetic digital tumor and concluded that the volume of a compact lesion can be determined with less than 10% error if there were at least 5 slices through the region of interest (18). To our knowledge, there is no published work that directly compares observer volumes based on repeat MRI with different pulse sequences, nor is there any work correlating MRI partial volume effect with dosimetric consequence in SRS plans. In this study, a set of phantoms was imaged with 4 different T1-weighted MRI sequences. The observed volumes were compared with true volume to evaluate inter-observer variation due to spatial resolution of MRI. SRS plans based on the observer volumes were generated and the correlation with the plan quality was evaluated.

## 2 Materials and methods

### 2.1 Phantoms

Four commercially available CT/MR phantom inserts were included in this study, all with one or two cavities fillable with MR-compatible solution to simulate tumor volume. Three are part of a STEEV phantom (CIRS, Norfolk, VA, USA) that simulate a 12.5 cm^3^ organic tumor (P1), a 25.0 cm^3^ organic tumor (P2), and a 3 cm diameter spherical tumor (P3), respectively. These cavities were filled with water doped with CuSO_4_5H_2_O. The 4th insert (P4), part of StereoPHAN (Sun Nuclear, Melbourne, FL, USA), has two identical cavities that were filled with mineral oil, each with a volume of 0.53 cm^3^. **Table 1** lists the four inserts. Select axial, coronal, and sagittal views through the inserts from one of the MRI series are included. The tumor volumes are surrounded by signal-free background on the MRI.

**Table 1:** Phantom inserts and simulated target shapes as shown in BRAVO MRI series.

Insert	Volume (cm^3^)	Axial	Coronal	Sagittal
P1	12.5	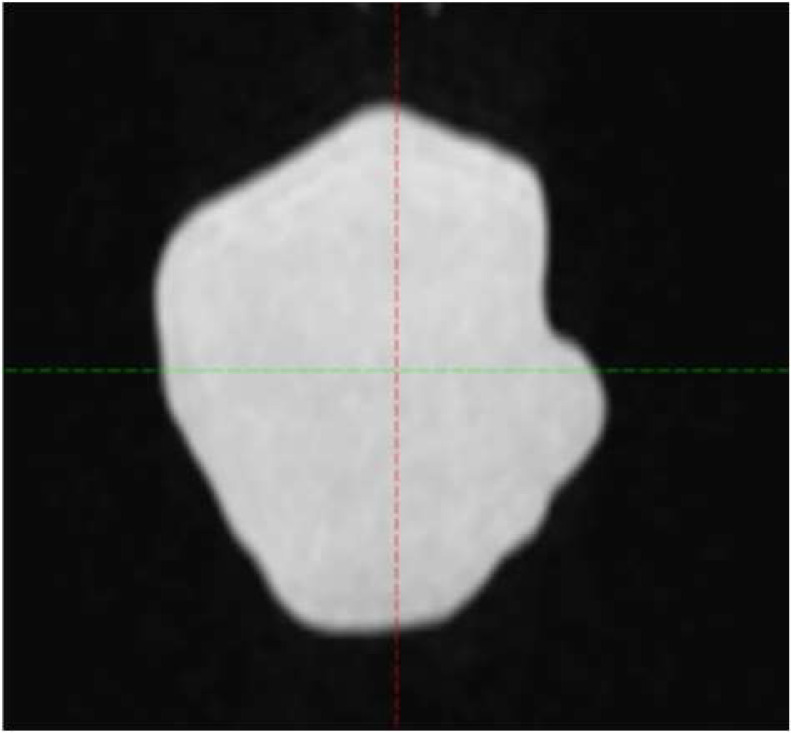	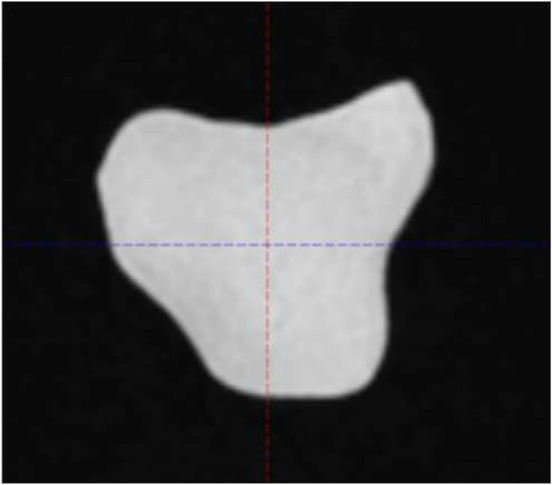	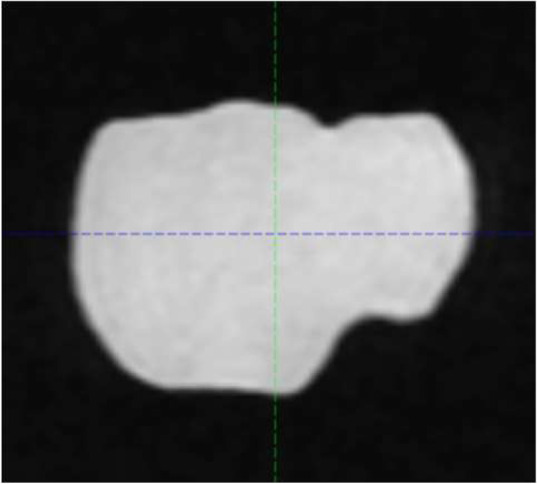
P2	25	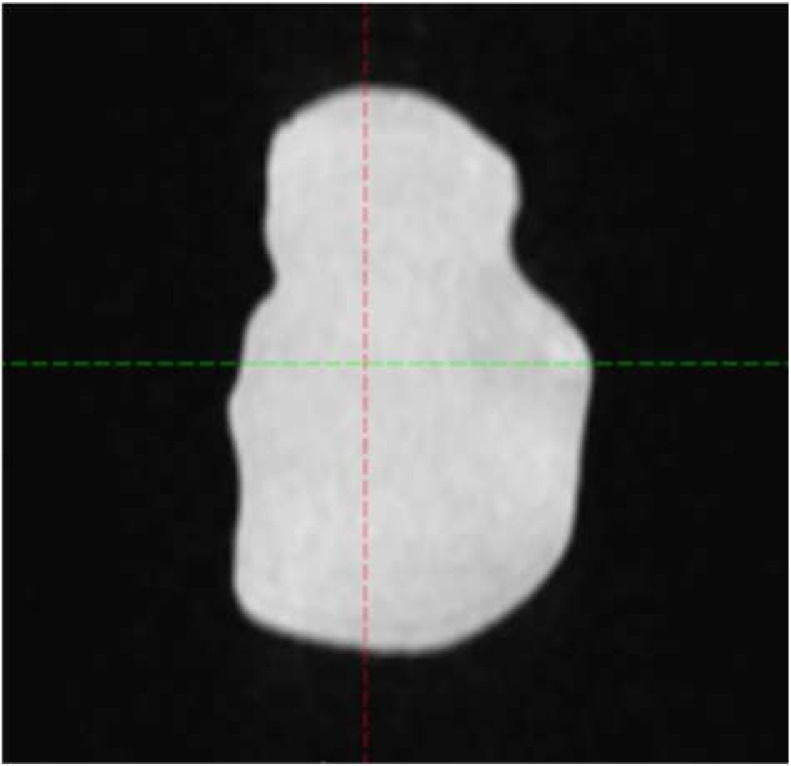	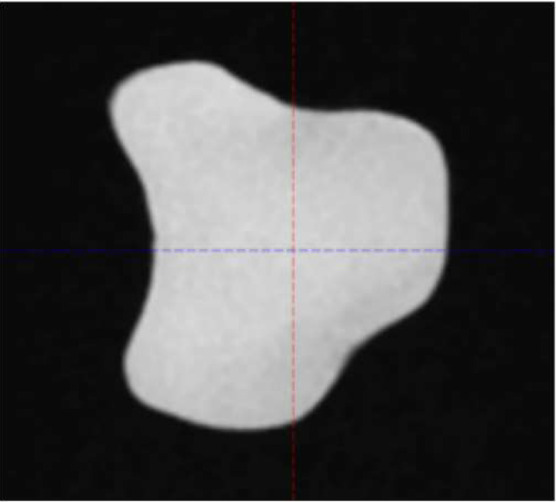	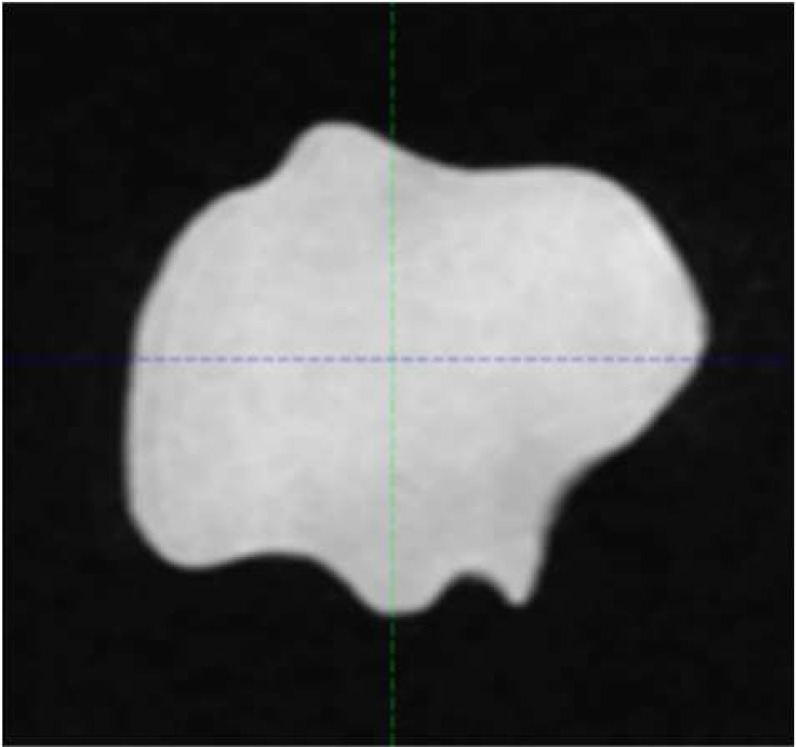
P3	14.1	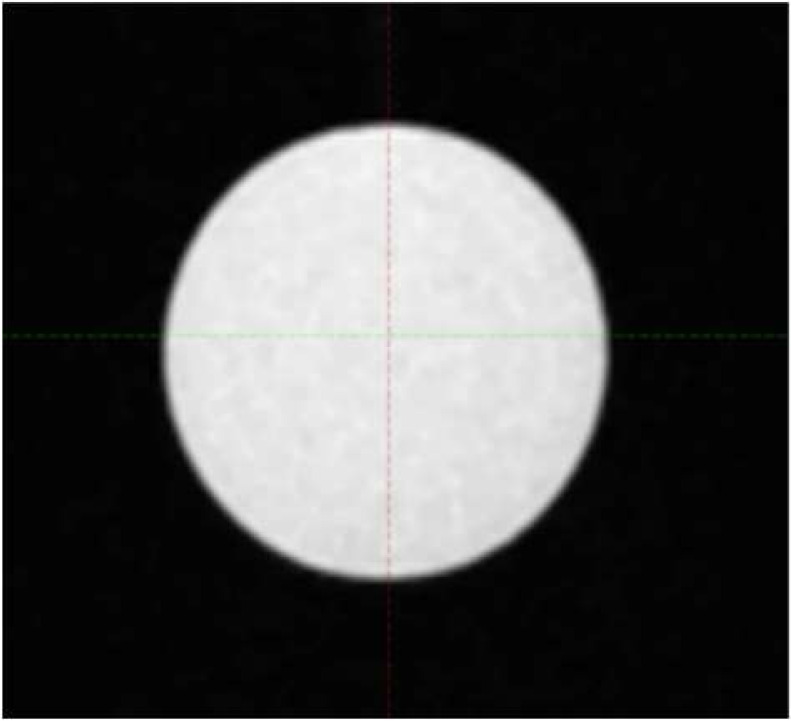	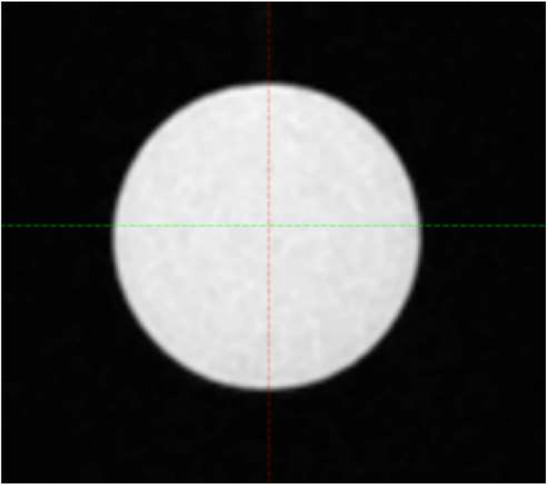	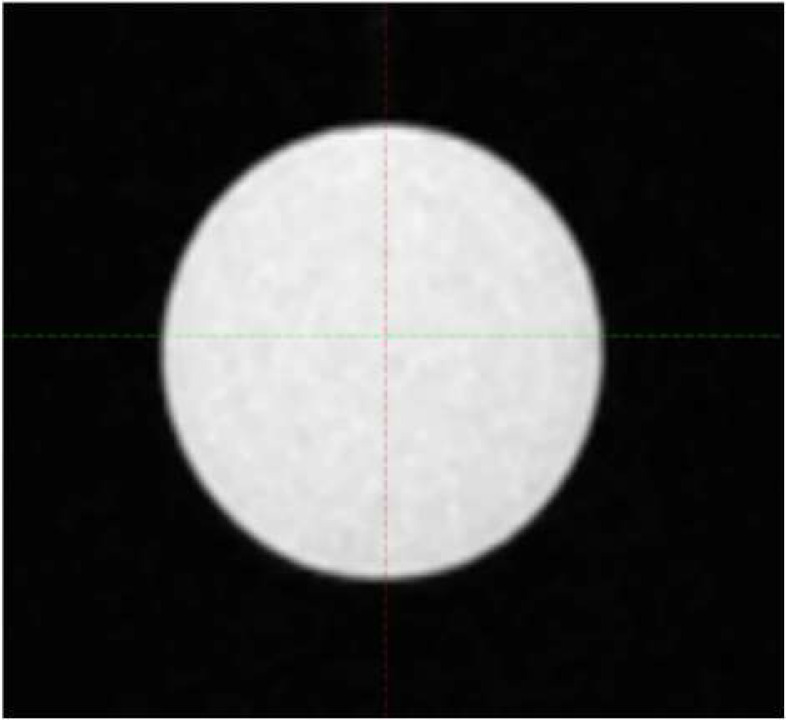
P4	0.53	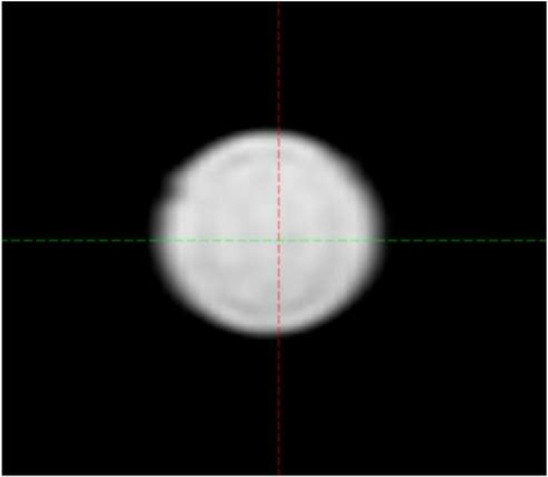	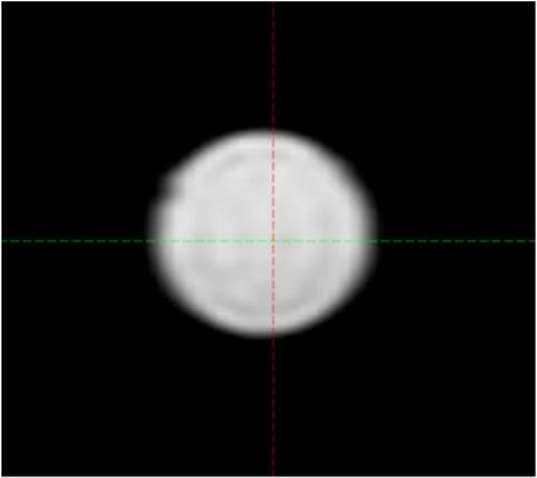	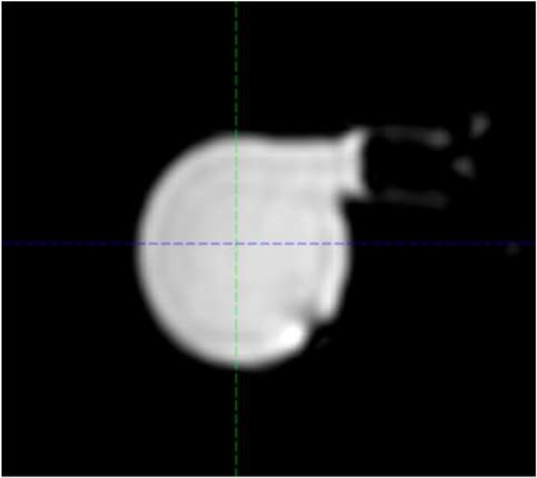

### 2.2 Imaging with CT and MR

All inserts were imaged on a Brilliance 64 CT scanner (Philips Medical Systems, Hamburg, Germany) with an axial pixel size of 0.23 × 0.23 mm^2^ and slice thickness of 0.65 mm. The images were imported in Eclipse (V15.6, Varian Medical Systems, Palo Alto, CA, USA). Target volumes were determined based on image thresholding with the absolute dimensions consistent with vendor specifications. These volumes are the gold standard to which the MR-based observer contours were compared.

The inserts were imaged with four different T1-weighted MR protocols on a Panorama 1.0T (Philips Medical Systems) and a Discovery MR750 3.0T (GE Healthcare, Chicago, IL, USA). **Table 2** lists the MRI protocols in order of increasing spatial resolution, and these represent the typical or best resolution in T1-weighted MRI encountered in our clinic. TFE and BRAVO sequences are both gradient echo 3D scan technique and provide high resolution images with near isotropic voxel dimensions. 2D multi-slice Spin Echo (SE) provides sub-millimeter pixel size in a pre-selected orientation but thicker slick thickness than the 3D scan images. To compensate for worse resolution cross-plane, each SE sequence was acquired in both axial and coronal orientations. The pair of images was presented to the observers as a set to aid determination of the target volumes in three dimensions. Four sets of MRs, i.e., SE5mm, SE3mm, TFE, and BRAVO, were acquired for each insert, resulting in 16 sets of MR images.

**Table 2:** MR protocols listed with increasing image resolution.

Scanner	MR Protocol	Resolution in axial plane or plane of acquisition (mm^2^)	Slice thickness (mm)
GE 3.0T	SE5mm (T1-Flair SE axial and coronal) TR = 2100 ms, TE = 8.3 ms, TI = 895 ms	0.4 × 0.4	5.0
Philips 1.0T	SE3mm (T1 SE axial and coronal) TR = 643.9 ms, TE = 15 ms	0.5 × 0.5	3.0
Philips 1.0T	TFE TR = 7.9 ms, TE = 3.9 ms, flip angle = 9°	0.9 × 0.9	1.25
GE 3.0T	BRAVO TR = 7.5 ms, TE = 3.2 ms, flip angle = 12°	0.4 × 0.4	0.5

### 2.3 Contouring

Four radiation oncologists experienced with cranial radiosurgery provided the contours in Eclipse (Varian Medical System, Palo Alto, CA). The contours were manually delineated on individual set of MR series without referencing other MRI or CT image sets. As described above, the true volume was determined based on CT. To compare observer contours with true volume, all observer contours were transferred from their respective MR series to the corresponding CT dataset through rigid image registration. The CT and all the true and observer contours were then exported to MIM (V6.8.7, MIM Software Inc. Cleveland, OH, USA) for the final analysis. The following metrics were included for comparison:

a. Dice similarity coefficient (DSC) (19): measures the overlap between observer contour, A, and ground truth, B. The values of DSC range from 0 (no overlap) to 1 (perfect overlap).


$$DSC=\frac{2\left(A\cap^{\;}B\right)}{A+B}$$

b. Ratio of observer volume to true volume (Ratio_Vol_).

c. Hausdorff distance (HD) (20): the maximum distance between contours A and B, or *max(h(A,B), h(B, A))*, where *h(A, B)* = $\underset{a\in A}{max}\underset{b\in B}{\;min}\mathrm{||}a-b\mathrm{||}$ , and ||*a*−*b*|| is the Euclidean distance.

d. Mean distance to agreement (MDA) (21): the average of all distances between points on contour A and contour B and vice versa.

The above parameters measure how close each observer contour is to true volume. The results on the observer contours from one MR sequence were compared with those from another MR sequence to demonstrate the effect of MR sequence resolution. Two-sided signed-rank tests were performed between SE5mm and SE3mm, between SE3mm and TFE, and between TFE and BRAVO. A value of *p *< 0.05 was pre-determined to indicate a significantly different result.

### 2.4 Dosimetric impact of observer contour variation

The true and observer volumes were all transferred to the STEEV anthropomorphic phantom CT dataset. Assuming the only organ-at-risk is the normal brain, a HyperArc plans (22) was generated with the planning target volume being one of the observer volumes. A total of 16 HyperArc plans were created based on the 16 observer volumes on insert P1 (P1-plans), and 24 HyperArc plans based on the observer volumes on P4 (P4-plans).

**Figure 1:**
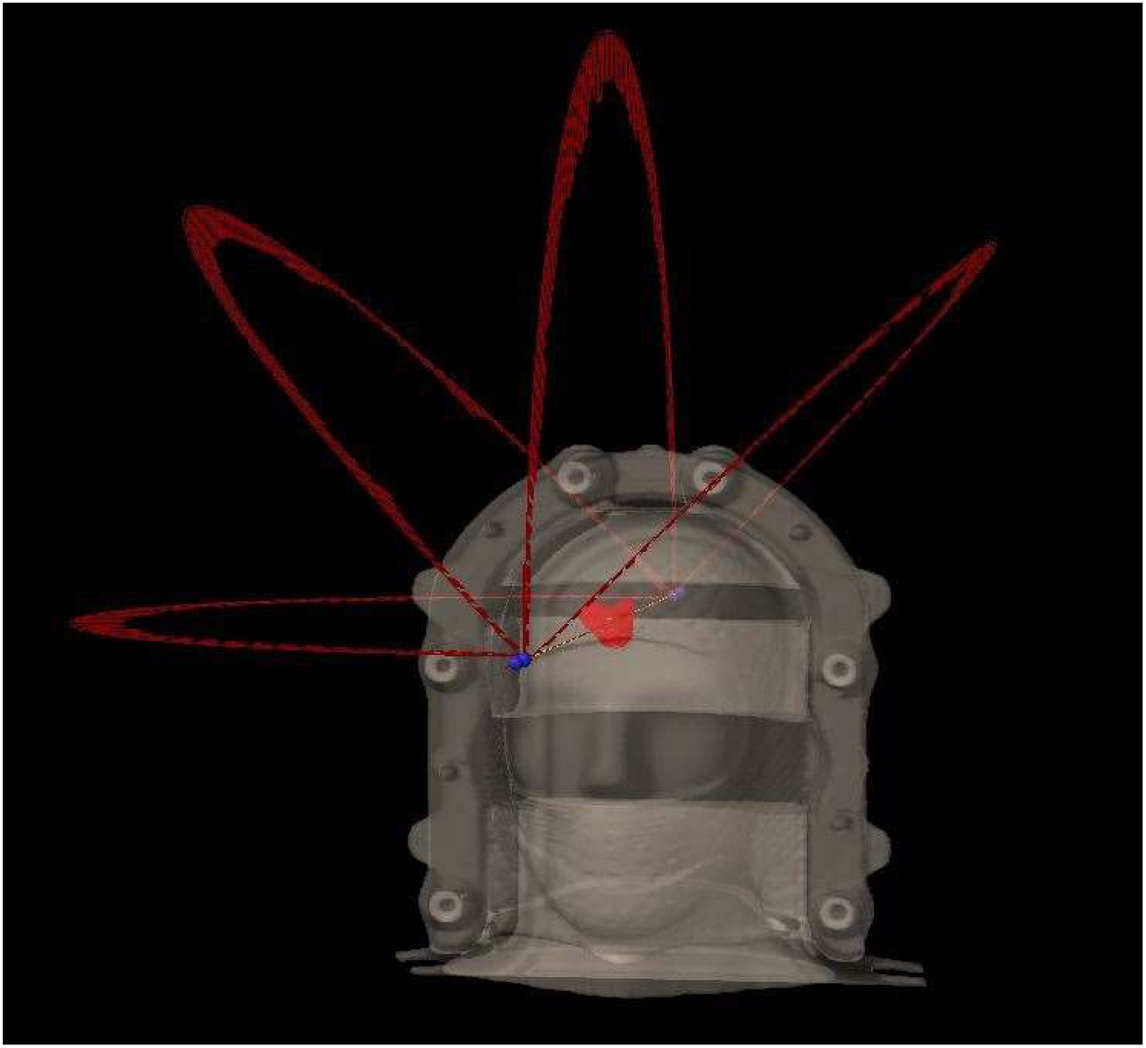
Typical beam arrangement of a HyperArc plan in this study, consisting of four non-coplanar 180° arcs at couch angles of 0, 45°, 90°, and 315°.

All the plans were generated in 6XFFF mode on an Edge with HD120 MLC (Varian Medical System, Palo Alto, CA), each consisted of four 180° arcs arranged at couch angles of 0, 45°, 270°, and 315°. A typical beam arrangement in a HyperArc plan is shown in **Figure 1**. The plans were optimized to provide optimal dose gradient and conformity and were normalized such that 100% prescription dose covered 99% planning target volume (V100% = 99%). The prescription was 27 Gy in 3 fractions to P1-plans and 18 Gy in single fraction to P4-plans, due to the target volume being close to 12.5 and 0.53 cm^3^, respectively.

The Paddick conformity index, CI_paddick_, is recommended in the International Commission on Radiation Units and Measurements (ICRU) report 91 (23) for reporting SRS treatments. It is defined as $CI_{paddick}=\;\frac{TV_{PIV}^{2}}{TV\times PIV}$ , where PIV is the volume of prescription isodose, TV is the target volume, and TV_PIV_ is the target volume covered by the prescription dose. An ideal value of CI_paddick_ is 1.0. Either under- or over-coverage of target volume leads to *CI_paddick_* being less than 1.0 and a decrease in plan quality.

In each of the HyperArc plans, *CI_paddick_* was evaluated using both the planning ( $CI_{paddick}^{planning}$ ) and true target volume ( $CI_{paddick}^{true}$ ). To evaluate the dosimetric impact due to the accuracy of planning target volume, the Pearson correlation coefficient, r, was calculated between $CI_{paddick}^{true}\;$ and *DSC* of the planning target volume. The correlation is considered strong if | *r* | > 0.7 and statistically significant if p < 0.05.

## 3 Results

### 3.1 Contouring

From the 4 observers and 4 inserts, 18 sets of contours were collected in each MR sequence. All observer contours were compared with the ground truth. The comparison to true volume as a function of MR sequence is presented in **Table 3**. All indices improved as the MRI spatial resolution increased from SE5mm, SE3mm, to TFE and BRAVO. The improvement of observer contours in DSC, HD, and MDA was statistically significant (p < 0.01). Thus, a higher MRI spatial resolution resulted in observer contours closer to ground truth.

**Table 3:** Comparison of observer delineated versus ground truth contours using DSC, HD, MDA, and Ratio_vol._.

MR Sequence	DSC	HD (mm)	MDA (mm)	Ratio_Vol_
SE5mm	0.86 ± 0.09 [0.63-0.94]	3.77 ± 1.96 [1.17-8.04]	0.75 ± 0.33 [0.27-1.44]	1.23 ± 0.34 [0.87-2.09]
SE3mm	0.91 ± 0.05 [0.79-0.96]	2.72 ± 1.22 [1.05-5.29]	0.47 ± 0.18 [0.24-0.93]	1.08 ± 0.19 [0.79-1.52]
TFE	0.94 ± 0.04 [0.85-0.97]	2.19 ± 1.28 [0.94-4.96]	0.31 ± 0.12 [0.13-0.56]	1.06 ± 0.13 [0.86-1.35]
BRAVO	0.96 ± 0.02 [0.92-0.98]	1.52 ± 1.28 [0.68-4.07]	0.18 ± 0.07 [0.10-0.31]	1.00 ± 0.07 [0.91-1.17]

### 3.2 Planning

**Figure 2:**
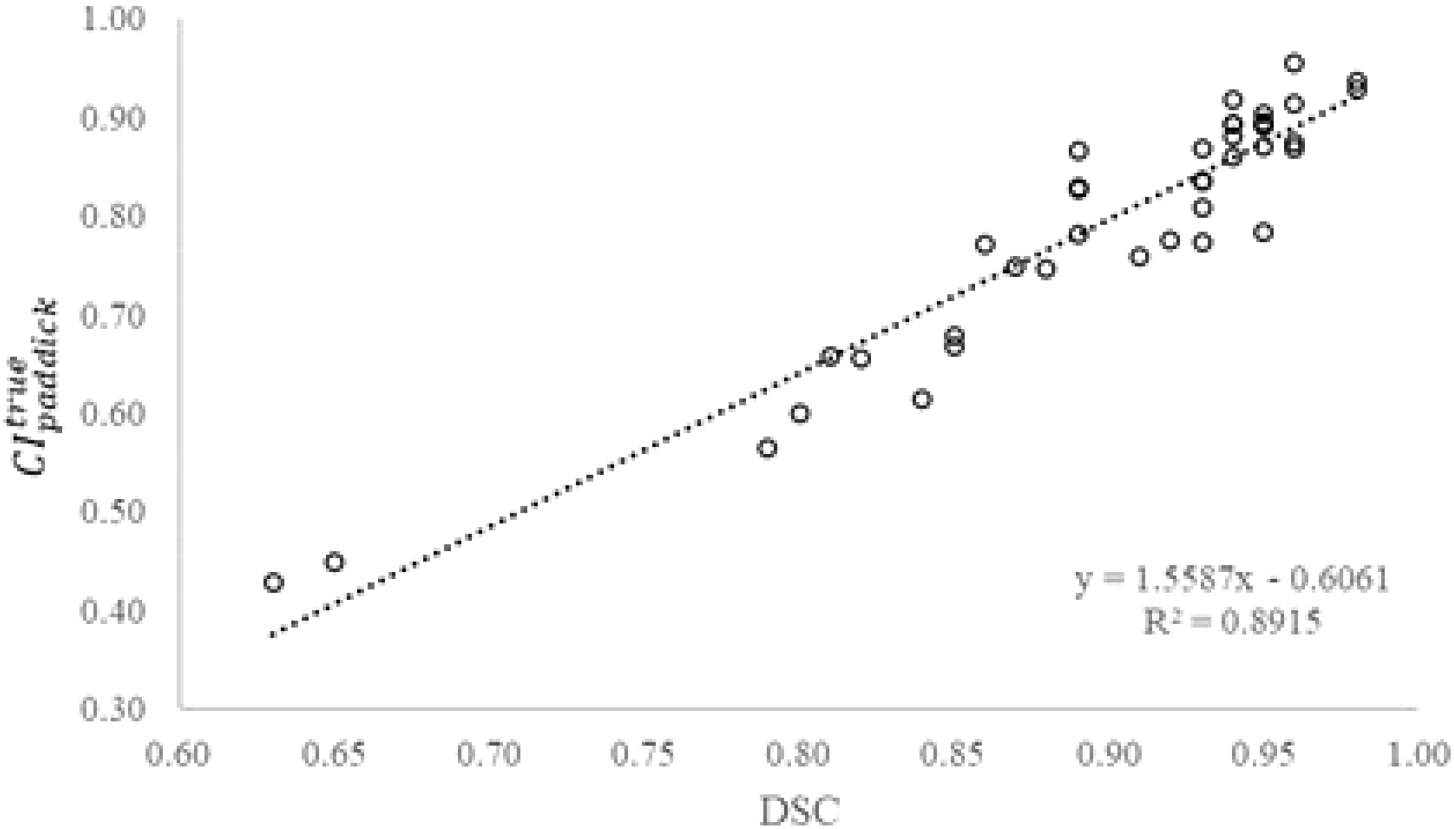
True Paddick conformity index ( $CI_{paddick}^{true}$ ) as a function of Dice similarity coefficient (*DSC*) of the planning target volume (N=40).

The 16 P1-plans were well matched in dose conformity and gradient based on the planning target volume. In particular, the value of $CI_{paddick}^{planning}$ in the 16 P1-plans was 0.92 ± 0.01 (range 0.91 to 0.93) according to the planning target volume. However, calculated with the true target volume, $CI_{paddick}^{true}$ was 0.88 ± 0.05 (range 0.75 to 0.96). Similarly, $CI_{paddick}^{planning}$ was 0.82 ± 0.02 (range 0.79 to 0.85) in the 24 P4-plans, while $CI_{paddick}^{true}$ degraded to 0.73 ± 0.13 (range 0.43 to 0.89). Combining the 16 P1-plans and 24 P4-plans, **Figure 2** shows a strong correlation between $CI_{paddick}^{true}$ and *DSC* of the planning target volume (r = 0.94, p< 0.00001). The positive correlation between $CI_{paddick}^{true}$ and *DSC* suggested that, as *DSC* decreases due to a greater deviation of planning target volume from true volume, $CI_{paddick}^{true}$ declines. Therefore, the accuracy of observer target volume directly impacted the quality of SRS plans.

## 4 Discussion

Although MRI offers superior soft tissue contrast, significant inter-observer variations have been noted in target delineation of brain tumors for SRS (24–26). Our study indicated that differences in image resolution in MRI sequences can contribute to inter-observer variation as demonstrated by parameters such as *DSC*, *HD* and *MDA* when comparing observer contours with ground truth. Higher resolution MRI leads to target volumes closer to ground truth and reduced spread in the parameters evaluated. Thus, finer resolution MRI allows more accurate and consistent target delineation among the observers.

**Figure 3:**
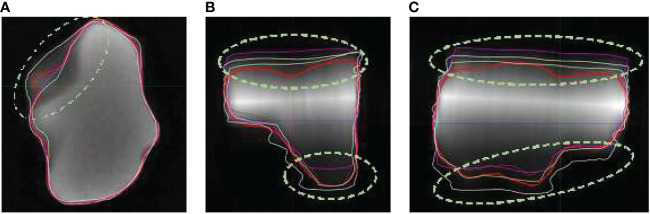
Observer contours on SE5mm MR sequence and true volume (red) on Phantom insert P1 in axial **(A)**, coronal **(B)**, and sagittal **(C)** views. The volumes are shown on SE5mm axial MR series with full range of window and level. Circled area showed significant deviation from true volume.

Both axial and coronal series were provided to the observers in the SE5mm and SE3mm sequences. The axial series has superior axial planar resolution, but coarse resolution in superior/inferior direction. The coronal series complements the axial series by providing superior resolution in coronal plane but coarse resolution in anterior/posterior direction. Combining information from the two series effectively allows the volume of a target to be defined accurately in three dimensions. Due to the subjectivity in combining information from axial and coronal series however, the superior and inferior portion of the volume could be either over- or under-represented, especially with irregular target volume shapes. In the clinical setting of cranial radiosurgery, the risk of over- or under-treating the superior or inferior portion of the disease increases when multi-slice 2D sequences with thick slices are used for SRS treatment planning. **Figure 3** compares true volume with the axial, coronal, and sagittal views of observer contours based on SE5mm sequence on phantom insert P1. The volumes were shown on the SE5mm axial series with full range of window and level. Besides the significant variation in observer contours at superior and inferior portions of the volume, large deviations were also noted where there is substantial change in tumor shape across plane (**Figure 3A**)

**Figure 4:**
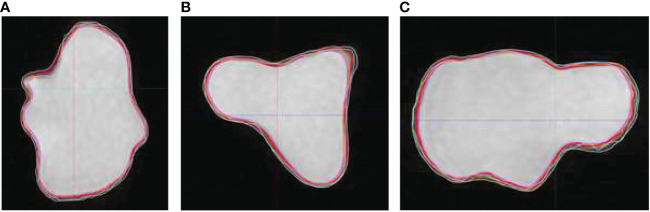
Observer contours on BRAVO MR sequence and true volume (red) on Phantom insert P1 in axial **(A)**, coronal **(B)**, and sagittal **(C)** views. The volumes are shown on BRAVO series with full range of window and level.

The other factor that contributes to the inter-observer variation was the difference in user window/level preferences and related impact on visualization of target boundaries. This factor leads to systematic over- or under-estimation of the target volume. **Figure 4** compares true volume with observer contours based on BRAVO sequence on phantom insert P1. Comparing to **Figure 3**, the agreement with true volume is much improved.

To avoid being influenced by other MRI series or CT images, the observers were instructed to delineate directly on the individual MR sequence. The volumes were then transferred on to the CT image set. All the transferred volumes were visually confirmed to be consistent with the original observer volumes on MRI. The CT image, true volume, and transferred observer volumes were then exported and analyzed in MIM. There could be potential differences in volume calculation and rendering between different systems (27, 28). To confirm fidelity of volumes following transfer from Eclipse to MIM, volumes reported by the two systems were compared, and the difference was negligible 0.01 ± 0.01 cc. Because all the analysis was carried out on one system, uncertainty due to variations in the handling of the volumes by different systems is minimized.

In this study, we simulated T1-weighted post-contract images using the MR and CT compatible inserts. The influence of other image series, such as pre-contrast T1-weighted, T2-weighted, fluid-attenuated inversion recovery image, etc, which might be useful for target delineation in SRS planning was not investigated. The simulated target volumes were well-defined and surrounded by signal-free background in the MR images. In a clinical brain MR image, it is likely that inter-observer variation would increase due to MR spatial resolution and other factors including image contrast and signal-to-noise ratio (SNR). The lesions included in this study were small and the MRIs were acquired with targets placed in the magnetic isocenter. For large lesions, or discrete lesions spread out over a large volume, any geometric distortion in the MRI could also lead to systematic deviation in user contours.

## 5 Conclusion

Significant improvements in target definition and reduced inter-observer variations were observed as the MR image resolution improved. Results imply that the highest resolution 3D MR sequences should be used to minimize potential errors in target definition, and multi-slice 2D sequences should be avoided. Even with high-resolution 3D MR sequences, care should be used with the window and level of the image for consistent target definition.

## Data availability statement

The raw data supporting the conclusions of this article will be made available by the authors, without undue reservation.

## Author contributions

YH was responsible for the research design, data collection and analysis, and manuscript preparation. EL, ES, MS, SS participated in data collection. All authors contributed to the article and approved the submitted version.
